# Five Shades of Grey: Exploring Quintary *m*-Sequences for More User-Friendly c-VEP-Based BCIs

**DOI:** 10.1155/2020/7985010

**Published:** 2020-03-10

**Authors:** Felix W. Gembler, Aya Rezeika, Mihaly Benda, Ivan Volosyak

**Affiliations:** Rhine-Waal University of Applied Sciences, 47533 Kleve, Germany

## Abstract

Responsive EEG-based communication systems have been implemented with brain-computer interfaces (BCIs) based on code-modulated visual evoked potentials (c-VEPs). The BCI targets are typically encoded with binary *m*-sequences because of their autocorrelation property; the digits one and zero correspond to different target colours (usually black and white), which are updated every frame according to the code. While binary flickering patterns enable high communication speeds, they are perceived as annoying by many users. Quintary (base 5) *m*-sequences, where the five digits correspond to different shades of grey, may yield a more subtle visual stimulation. This study explores two approaches to reduce the flickering sensation: (1) adjusting the flickering speed via refresh rates and (2) applying quintary codes. In this respect, six flickering modalities are tested using an eight-target spelling application: binary patterns and quintary patterns generated with 60, 120, and 240 Hz refresh rates. This study was conducted with 18 nondisabled participants. For all six flickering modalities, a copy-spelling task was conducted. According to questionnaire results, most users favoured the proposed quintary over the binary pattern while achieving similar performance to it (no statistical differences between the patterns were found). Mean accuracies across participants were above 95%, and information transfer rates were above 55 bits/min for all patterns and flickering speeds.

## 1. Introduction

Maximum length sequences (*m*-sequences) are special pseudorandom binary sequences that have been used in various research fields including encryption, signal recovery, and brain-computer interface (BCI) [1–3].

A BCI is an interface between a user's brain and a computer; it translates the brain activities into commands allowing the control of external devices without muscle activity [4]. The BCI paradigm based on code-modulated visual evoked potentials (c-VEPs) interprets the responses to rapidly flickering patterns corresponding to special code sequences [5–8]. Each c-VEP target is coded with an individual sequence, where bits are mapped to different contrasts. To encode targets on computer monitors, usually black and white patterns are used [9].

The brain responses to these patterns (the c-VEPs) can be recorded via electroencephalography (EEG). A typical c-VEP application is a communication tool, where a target letter fixated by the user is determined via template matching [10].

Although c-VEP spelling applications can achieve high communication speeds (around 20 error-free characters per minute [5]), some issues with regard to user friendliness need to be addressed.

A key aspect in terms of usability is the flickering speed. In general, the number of bit flips per second impacts the classification accuracy [11]. Numerous BCI studies investigated stimulus choice for the steady-state VEP (SSVEP) approach, where targets are coded with distinct frequencies [12, 13]. According to Herrmann [14], brain responses of up to 90 Hz can be recognized in EEG recordings. Low-frequency and medium-frequency sets between 6 and 30 Hz are predominantly used for spelling applications in SSVEP research [10, 15] as they elicit large SSVEP amplitudes.

However, BCI users may perceive low flickering speed as annoying and tiring [16, 17]. This also applies to flicker patterns based on *m*-sequences. Stimulus-induced fatigue reduces the applicability of these systems. Moreover, the low-frequency flicker patterns may trigger photosensitivity-based epileptic seizures [17].

Because of these problems, high-frequency BCI applications have been developed [13, 16]. For example, Chen et al. [13] implemented a 45-target SSVEP BCI speller using high-frequency stimuli (ranging from 35.6 to 44.4 Hz). The authors reported a promising average information transfer rate (ITR) of 61 bits/min. Armengol-Urpi and Sarma [18] integrated high-frequency stimuli (42, 43, 44, and 45 Hz) in a virtual reality menu navigation tool. The authors stated that users reported a satisfactory overall experience as the flickering did not cause annoyance. Even higher, imperceptible flickers around 60 Hz have also been tested: Sakurada et al. [16] used three LED stimuli (61, 63, and 65 Hz) and reported an average accuracy of 90% while eliminating visual fatigue. More recently, Jiang et al. [19] used four phase-shifted 60 Hz stimuli presented on a 240 Hz monitor.

For c-VEP BCIs, the flickering speed can be manipulated by changing the monitor refresh rate. When using standard 60 Hz monitors, the stimulus duration of a 63 bit *m*-sequence is 63/60 = 1.05 s, a time window that is reasonably fast while still sufficiently long for reliable classifications. Higher refresh rates allow for higher flickering rates, which can potentially improve user friendliness. However, the target stimuli might be harder to distinguish from other targets due to the shorter lag between consecutive targets. Previous research indicates that c-VEP stimuli generated with a 120 Hz refresh rate yield good performance [20–22], but with a 240 Hz setup, a performance drop has been observed [23]. In terms of bit flips per second, the 240 Hz generated *m*-sequence is comparable to a 59 Hz SSVEP stimulus. Due to the sequences of up to 6 consecutive identical bits, the flickering pattern generated by the *m*-sequence is still visually perceivable.

Beside higher flickering rates, research on SSVEP-BCIs has found other methods to reduce discomfort induced by the flickering. For example, with the sinusoidal stimulus modulation method [24], which is realised by varying the luminance each frame, more subtle sine-shaped stimulus patterns can be realised. Recently, we compared the stimulus presentation paradigms SSVEP and c-VEP in terms of system performance and user friendliness [25]. While c-VEP slightly outperformed SSVEP in terms of offline accuracy, SSVEP was rated as the more user-friendly approach (thanks to the more subtle sinusoidal stimulus presentation).

Due to the binary stimulation pattern of the *m*-sequence, the visual stimuli switch between two colours (most commonly black and white). Other code patterns could offer a more subtle stimulation while maintaining good autocorrelation. Recently, Shirzhiyan et al. [26] employed chaotic codes generated from a one-dimensional logistic map. While there was no significant difference in the classification accuracies in comparison with conventional *m*-sequences, the chaotic code reduced subjective fatigue.

**Figure 1:**
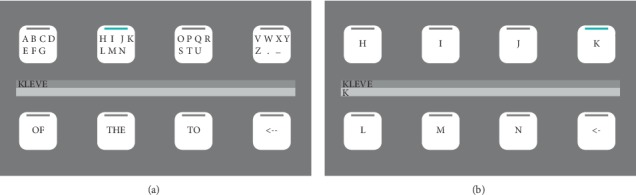
Graphical user interface of the c-VEP-based eight-target speller. Each target corresponded to a lagged version of an m-sequence. By selecting a group of letters (e.g., H–N), a second layer containing individual letters was displayed. In the example, the letter K was selected. In addition to letter groups, the first layer of the interface also presented three word suggestions based on an integrated dictionary function. The lower right target represented an undo function. Feedback was given to the user by enlarging the selected target for 100 ms, voicing the corresponding letter, and adding it to the text output field in the centre of the screen.

In this study, quintary (base 5) *m*-sequences are explored. Instead of switching between black and white, the flickering targets go to five different shades of grey. We compared the BCI performance of the conventional binary and the proposed quintary pattern with refresh rate setups of 60, 120, and 240 Hz. The six different code patterns were tested with 18 participants using an earlier-developed spelling application [27, 28] that allows for the selection of letters in two steps (see Figure 1).

## 2. Methods

In the following, the generation of the binary and quintary *m*-sequence patterns and the respective stimulus designs are explained. Following that, details about the signal classification, the spelling application, and the experimental protocol are provided.

### 2.1. Participants

Eighteen nondisabled participants were recruited for this experiment, eight females and ten males (average age 24.3 years, SD 2.8, ranging from 18 to 29). All of them had normal or corrected-to-normal vision. This research was approved by the Ethical Committee of the Medical Faculty of the University of Duisburg-Essen. Before the experiment, the participants were informed about the purpose, risks, and experimental protocol of the study. The participants gave informed consent in accordance with the Declaration of Helsinki and were informed that they could opt out of the study without providing reasons at any time. The information needed for the analysis of the experiments was stored anonymously. All participants received a financial reward for taking part in the experiment.

### 2.2. Hardware

Stimulus presentation and signal identification operated on the same computer, Dell Precision 3630 Tower, equipped with an NVIDIA GeForce GTX 1080 graphics card running Microsoft Windows 10 Education on an Intel processor (Intel Core i7-8700K @ 3.70 GHz). The c-VEP targets were presented on a liquid crystal display screen (Acer Predator XB252Q, 1920 × 1080 pixels, 240 Hz refresh rate). For signal acquisition, an EEG amplifier (g.USBamp, Guger Technologies, Graz, Austria) was used, employing all its 16 signal channels, which were placed according to the international 10/5 system of electrode placement (see, e.g., [29]): P_Z_, P_3_, P_4_, P_5_, P_6_, PO_3_, PO_4_, PO_7_, PO_8_, POO_1_, POO_2_, O_1_, O_2_, O_Z_, O_9_, and O_10_. The reference electrode was placed at C_Z_ and the ground electrode at AF_Z_. The standard abrasive electrolytic electrode gel was applied between the electrodes and the scalp to bring impedances below 5kΩ during the preparation phase. A bandpass filter (between 2 and 100 Hz) and a notch filter (around 50 Hz) were applied. The sampling rate of the amplifier was set to 600 Hz.

### 2.3. Generation of *m*-Sequences

**Figure 2:**
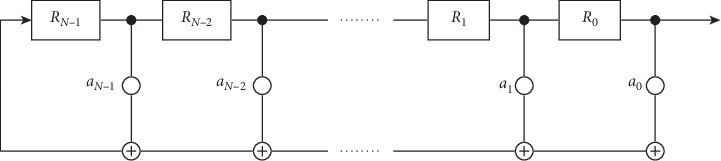
Generic linear-feedback shift register (LFSR). For the generation of a p-ary m-sequence of order N, the register consists of memory cells *R*_*i*_, *i*=0,…, *N* − 1, each holding a p-ary digit. The cells are controlled by a timing clock. At each pulse of the clock, the current values of the register cells are shifted to the next cell. To determine the next value in the leftmost register cell *R*_*N*−1_, the current register values are multiplied by weights *a*_*i*_ and then added together using modulo p arithmetics. The current value in the rightmost register cell *R*_0_ is appended to the output sequence.

A maximal-length sequence (*m*-sequence) is a periodic sequence with a noise-like waveform that can be generated using a linear-feedback shift register (LFSR) [30, 31] (see Figure 2). LFSRs are special shift registers, consisting of *N* memory cells (also called stages) labelled *R*_*N*−1_,…, *R*_1_, *R*_0_. The input digit stored in the cell *R*_*N*−1_ is the value of a linear function *f* that performs modulo *p* additions with a weighted subset of the register entries.

The memory stages of the LFSR are controlled by a timing clock. At each pulse of the clock, the state of each stage is shifted to the next stage. The entry in the cell *R*_*i*_ is passed to the cell *R*_*i*−1_, *i*=*N* − 1,…, 1. The entry in the stage *R*_0_ (the rightmost register) determines the output of the LFSR. The sequence of output bits is called the output stream of the LFSR.

A *p*-ary code of length *N* can assume *p*^*N*^ values. However, the period of the code produced by the LFSR can have a maximal length of at most *p*^*N*^ − 1. In this case, the LFSR cycles through all states except for the case where all digits are zeros. If all digits were zeros, it could not be used as a code sequence for stimuli, as there would be no state changes, and thus no brain response evoked by the stimuli. The output stream of maximal length is an *m*-sequence.

In Figure 2, a generic LFSR is displayed. The bit positions that influence the next state (weights *a*_*i*_ ≠ 0) are called taps. The combination of the register pins can also be expressed in the finite field arithmetic as the modulo *p* polynomial, which is referred to as a generator polynomial or feedback polynomial:

(1) $$\begin{array}{c} G\left(X\right)=X^{N}-\sum_{j=0}^{N-1}a_{j}X^{j}, \end{array}$$

where the coefficients *a*_*i*_ ∈ {0,1,…, *p* − 1} correspond to the weight of the register pin *R*_*i*_, *i*=0,…, *N* − 1.

An LFSR must be initialised with a so-called seed which describes the *N* initial digits of the register cells. The seed and the generator polynomial uniquely determine the resulting sequence.

If the LFSR is represented by a primitive polynomial and initiated with a nonzero seed, it will generate an *m*-sequence [32].

The binary *m*-sequence, *b*_1_, used in the experiment was determined with the generator polynomial *x*^6^+*x*^5^+1 (corresponding to weights *a*_0_=1, *a*_1_=*a*_2_=*a*_3_=*a*_4_=0, and *a*_5_=1) and the seed (*R*_5_, *R*_4_, *R*_3_, *R*_2_, *R*_1_, *R*_0_)=(1,1,0,1,0,1). The quintary *m*-sequence, *q*_1_, used in the experiment was determined with the generator polynomial *x*^3^+3*x*+2=*x*^3^ − 2*x* − 3 (corresponding to the weights *a*_2_=0, *a*_1_=2,  and *a*_0_=3) and the seed (*R*_2_, *R*_1_, *R*_0_)=(0,3,0). The period lengths, and thus the length of the *m*-sequence, were 2^6^ − 1=63 for the binary pattern and 5^3^ − 1=124 for the quintary pattern.

**Figure 3:**
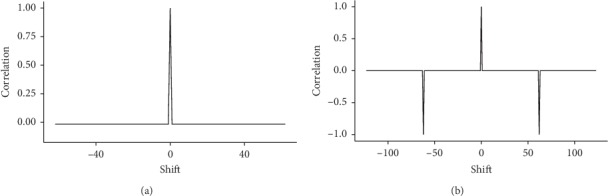
Cyclical autocorrelation function for a (a) binary 63-bit m-sequence and (b) quintary 124-digit m-sequence. Both autocorrelation functions are normalized so that the peak value is 1.

The *m*-sequences have a number of desirable properties (see, e.g., [33]). For BCIs, the most interesting feature is the autocorrelation function. For the binary sequence, a single peak at 0 can be observed. The values of the function are equal to 1 at …, −2*n*, −*n*, 0, *n*, 2*n*,…, and the correlation coefficient is 1/n in every other case, where n refers to the period length of the sequence, where *n* = 63 (i.e., binary 63-bit m‐sequence). It should be noted that the quintary *m*-sequence has two phase values for which the sequences are anticorrelated (see also [3]). These shifts are avoided in the implementation of the BCI. For the binary and quintary *m*-sequences used in the experiment, the autocorrelation functions are displayed in Figure 3.

### 2.4. Stimulus Design

To test the code sequences in an online spelling scenario, we implemented them into a spelling application with eight targets (230 × 230 pixels) which were arranged as a 2 × 4 stimulus matrix (see Figure 1). Each target corresponded to one of the *K*=8 code sequences.

For the binary flickering paradigm, *b*_1_ was generated as described in the previous section and *b*_2_, *b*_3_,…, *b*_*K*_ were generated by employing left circular shifts on *b*_1_ of 4 · 1,4 · 2,…, 4 · (*K* − 1). For the quintary flickering paradigm, the eight codes *q*_*i*_, *i*=1,…, *K*, were generated analogously.

The flickering patterns were modulated utilising alpha blending [34]. The process of alpha blending allows for transparency effects in computer graphics by applying a convex combination of two colours (a translucent foreground colour and a background colour). Using alpha blending, the translucent foreground colour of the stimulus (here white) was combined with the background colour (here black), yielding a blended colour (here different shades of grey). The degree of translucency, *α*, ranges from 0.0 to 1.0. When the foreground colour is completely transparent (i.e., *α*=0), the combined colour is the background colour (here black). On the contrary, if the foreground colour is completely opaque (i.e., *α*=1), the combined colour is the foreground colour (here white).

The degree of translucency of the stimuli was updated every frame; the values for *α* were derived from the code pattern. In case of the binary *m*-sequences, *α* was set to 0 or 1 in accord with the binary code sequence yielding a black and white pattern. For the quintary *m*-sequences, the quintary digits 0, 1, 2, 3, and 4 were mapped to the corresponding *α*-values 0, 0.25, 0.5, 0.75, and 1, yielding a pattern that goes through five shades of grey.

The update rate and therefore the speed of the flickering pattern are dependent on the monitor refresh rate. At high vertical refresh rates, a more subtle visual stimulation can be achieved. Here, for both code patterns, update rates of 60, 120, and 240 Hz were tested; the stimulus colour was updated every $16\text{.}\bar{6}$, $8\text{.}\bar{3}$, and $4\text{.}1\bar{6}$ ms, respectively.

### 2.5. Experimental Protocol

Each participant took part in six sessions using the two flickering patterns at three different update rates, 60 Hz, 120 Hz, and 240 Hz. The order was binary at 60 Hz, quintary at 60 Hz, binary at 120 Hz, quintary at 120 Hz, and binary at 240 Hz, quintary at 240 Hz, for half of the participants, and quintary at 60 Hz, binary at 60 Hz, quintary at 120 Hz, binary at 120 Hz, and quintary at 240 Hz, binary at 240 Hz, for the other half. In between sessions, participants took a small break. Each session consisted of a training and a copy-spelling phase. Participants sat in a comfortable chair approximately 70 cm away from the screen which presented the 8-target interface (arranged as a 2 × 4 matrix, see Figure 1), showing numbers 1–8 in the training phase and a letter grid in the copy-spelling phase.

**Figure 4:**
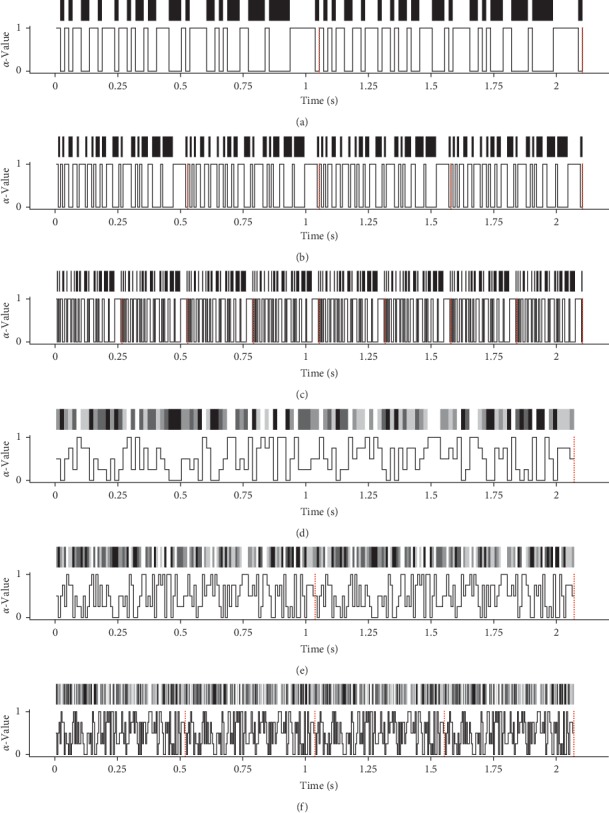
*α*-Values of the stimulus object for the six tested flickering patterns. Displayed are the *α*-values derived from binary and quintary code patterns at different monitor refresh rates. The *α*-values range from 0 to 1: 0 denotes “fully transparent” and 1 denotes “fully opaque.” The red line indicates the end of a full stimulation cycle. (a) Stimulus pattern of the binary 63-digit m-sequence, refresh rate 60 Hz, 2 cycles. (b) Stimulus pattern of the binary 63-digit m-sequence, refresh rate 120 Hz, 4 cycles. (c) Stimulus pattern of the binary 63-digit m-sequence, refresh rate 240 Hz, 8 cycles. (d) Stimulus pattern of the quintary 124-digit m-sequence, refresh rate 60 Hz, 1 cycle. (e) Stimulus pattern of the quintary 124-digit m-sequence, refresh rate 120 Hz, 2 cycles. (f) Stimulus pattern of the quintary 124-digit m-sequence, refresh rate 240 Hz, 4 cycles.

For the generation of c-VEP templates, labelled responses for every stimulus were recorded in the training phase, where all eight targets were presented simultaneously to the user. For each of the eight targets, several trials were recorded. In this respect, the training phase was grouped into *n*_*b*_ = 6 blocks, where 6 · 8 = 48 trials were collected in total. For the binary pattern, each trial lasted 2.1 s; the stimulation cycle repeated 2, 4, and 8 times for the 60, 120, and 240 Hz setups, respectively. Analogously, for the quintary pattern, each trial lasted $2\text{.0}\bar{6}$ s; the stimulation cycle repeated 1, 2, and 4 times for the 60, 120, and 240 Hz setups. The different flickering patterns are illustrated in Figure 4. The trials were stored as an *m* × *n* matrix, where m denotes the number of recording EEG electrodes (here *m* = 16) and n denotes the number of sample points (here *n* = 2.1 · *F*_*s*_ = 1260 and *n* = 2.06 · *F*_*s*_ = 1240 samples for binary and quintary patterns) samples, for binary and quintary patterns).

The box, at which the user was needed to gaze, was outlined by a green frame. The boxes were highlighted in sequence (from the upper left to the lower right). After each trial, the flickering paused for 1 s. After each block, the user could rest for a longer time, until he or she initiated the next recording block by pressing the space bar on the keyboard.

After each training phase, participants filled out a brief questionnaire. The subjective impressions of the flickering patterns were assessed with two 7-point Likert scales: (1 = relaxing, 7 = exhausting) and (1 = comfortable, 7 = annoying), where the points 2–6 were left unlabelled.

In the online session, a brief familiarisation run was conducted, where participants learned how to use the speller. Thereafter, a copy-spelling task was performed. Misclassifications needed to be corrected by gazing at the box representing the UNDO function. The copy-spelling task was to spell the word POWERFUL. In this phase, the gaze-shifting phase was 2 s, giving the participant enough time to identify the location of the next character. (During this gaze-shifting phase, the flickering and data recording paused). The entire experiment lasted approximately 1 h.

### 2.6. Signal Classification

A template-matching method using spatial filters generated via canonical-correlation analysis (CCA) was used for online signal classification [27]. A filter bank design was used to increase the discrimination of targets [35] further. On the basis of the training data, templates were calculated by averaging over the target-specific trials. In addition to this, for each target, a CCA-based spatial filter *w*_*i*_ was determined as described, e.g., in [28].

This was done for *M*=3 different filter banks; in this regard, *M* bandpass filters (described in the following section) were applied to the recorded trials, resulting in weights *w*^(*m*)^ ∈ *ℝ*^*m*^ and templates *X*_*i*_^(*m*)^ ∈ *ℝ*^*m*×*n*^, *i*=1,…, *K*, for *m*=1,…, *M*.

The three filter banks were designed using 8th-order Butterworth bandpass filters. The upper and lower cutoffs were set as follows:

1. The first subband covered the alpha, beta, and gamma bands (a bandpass filter between 8 and 60 Hz was applied)
2. The second subband covered the beta and gamma bands (a bandpass filter between 12 and 60 Hz was applied)
3. The third subband covered the gamma band (a bandpass filter between 30 and 60 Hz was applied)

For classification, ensemble correlations between spatially filtered reference signals and the spatially filtered EEG data buffer were calculated for each subband (*m*=1,…, *M*) independently. This yielded a set of correlation coefficients:

(2) $$\begin{array}{c} {\tilde{\lambda }}_{k}^{\left(m\right)}=\rho \left(\left[\begin{array}{c} Y^{T}w_{1}^{\left(m\right)}\\ \vdots \\ Y^{T}w_{K}^{\left(m\right)} \end{array}\right],\left[\begin{array}{c} X_{k}^{\left(m\right)T}w_{1}^{\left(m\right)}\\ \vdots \\ X_{k}^{\left(m\right)T}w_{K}^{\left(m\right)} \end{array}\right]\right), \end{array}$$

which were calculated for all classes *k*=1,…, *K* and averaged across the number of filter banks:

(3) $$\begin{array}{c} \lambda_{k}=\frac{1}{M}\overset{M}{\underset{m=1}{\sum }}{\tilde{\lambda }}_{k}^{\left(m\right)},\;k=1,\ldots ,K. \end{array}$$

To identify the intended target, the class label *C* was determined as

(4) $$\begin{array}{c} C=\underset{k=1,\ldots ,K}{\arg\max}\lambda_{k}. \end{array}$$

For the online classification, a sliding window mechanism was implemented as described in [25]. The amplifier transferred the EEG data in blocks of 30 samples per channel, which were collected in a buffer. The number of columns, *n*_*y*_, of the buffer Y changed dynamically when new data were added each calculation interval (*n*_*y*_ incrementally increased by 30 samples until *n*_*y*_=*n*). After a new block was received, a class label was calculated using submatrices from the templates (containing only the first *n*_*y*_ columns). A system output was only produced, if a threshold criterion was met: the distance between the highest and the second highest correlation needed to exceed 0.15; for some participants, this threshold was adjusted slightly during the familiarisation to increase accuracy. If this threshold criterion was met, the output was produced, the data buffer *Y* was cleared, and a gaze-shifting period of two seconds followed. If the criterion was not met, further data were added to the buffer. In case *n*_*y*_=*n*, old data were shuffled out.

## 3. Results

In the following, the results from the evaluation of the online spelling performance and the questionnaire are presented; Table 1 provides an overall summary of the results. The BCI performance was evaluated by comparing ITR and classification accuracy. The significance levels of the differences between the binary and quintary patterns were evaluated using paired *t*-tests. We used Wilcoxon signed-rank tests and Friedman's analysis to evaluate the questionnaires.

### 3.1. Offline Performance Evaluation

**Figure 5:**
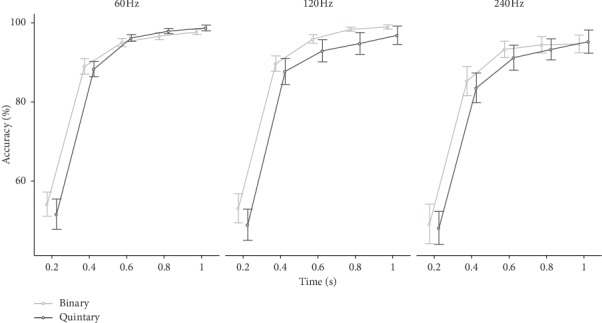
Offline results for the binary and quintary m-sequences. Mean accuracies across all 18 participants for different classification time windows are provided. The error bars indicate standard errors of the means.

The offline classification accuracy of the binary and quintary flickering paradigms was compared offline via leave-one-out cross-validation (see, e.g., [36]). All but one of the recorded blocks (each containing eight trials) was used for the training, and the left-out block was used as validation data. The cross-validation was repeated *n*_*b*_ times; each recording block was used once as validation data, and the resulting accuracies were averaged. For the performance analysis, the process was conducted for different classification time windows up to 1 s.  Figure 5 presents the mean classification accuracies for all tested patterns.

For the time window of 1 s, the mean (SD) classification accuracies for the binary flickering pattern were 97.7 (2.76) %, 99.0 (3.3)%, and 94.7 (9.6)% for the 60 Hz, 120 Hz, and 240 update rates, respectively; for the quintary flickering pattern, accuracies were 98.7 (3.1)%, 96.9 (9.8)%, and 95.3 (12.4)%. Neither for the 60 and 120 Hz refresh rates nor for the 240 Hz refresh rate, significant differences between the binary and quintary patterns were found according to paired *t*-tests (*p* > 0.05).

In general, the accuracy achieved with the fastest flickering rate (using the 240 Hz refresh rate) was lower in comparison with those of the 60 and 120 Hz refresh rates. No statistical differences between the binary and quintary patterns can be observed.

### 3.2. Online Spelling Performance Evaluation

**Figure 6:**
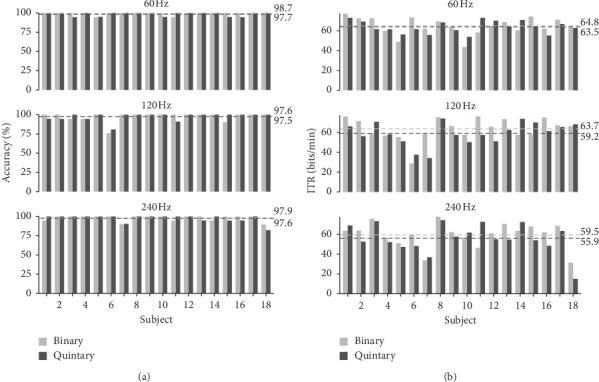
Online results for the binary and quintary m-sequences. All 18 participants spelt the word POWERFUL with different flickering speeds. (a) Accuracies and (b) information transfer rates (ITRs) for the 60 Hz, 120 Hz, and 240 Hz refresh rate setups are shown. The dashed lines indicate the mean values across all participants.

Figure 6 shows the individual performance in the online experiment. The commonly used ITR and classification accuracies were calculated. The ITR in bits/min, *B*_*m*_, is given as follows:

(5) $$\begin{array}{c} B_{m}=\frac{\log_{2}K+p\log_{2}p+\left(1-p\right)\log_{2}\left(\left(1-p\right)/\left(K-1\right)\right)}{t/60}, \end{array}$$

where *K* denotes the number of classes (here *K*=8), *p* denotes the classification accuracy which is calculated as correctly classified selections divided by the total number of selections, and *t* denotes the average time to make a selection (in s). An online calculation tool for the ITR can be found at https://bci-lab.hochschule-rhein-waal.de/en/itr.html.

**Figure 7:**
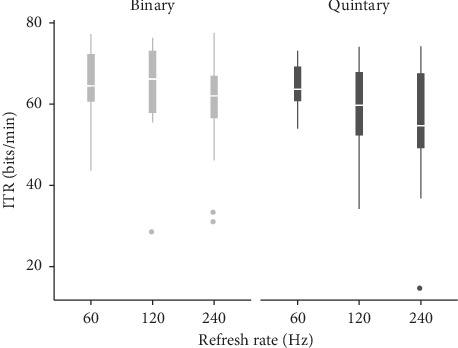
Online ITRs for the binary and quintary m-sequences. The values achieved by the 18 participants in the copy-spelling phase are shown per refresh rate. In the box plots, outliers (data points outside 1.5 times the interquartile range) are located outside the “whiskers.”

All participants completed the task for all six tested flickering patterns. The average (SD) online classification accuracies for the binary flickering pattern were 99.4 (1.85) %, 97.6 (6.0)%, and 97.9 (3.6)% for the 60 Hz, 120 Hz, and 240 Hz update rates, respectively; for the quintary flickering pattern, accuracies were 98.5 (2.5)%, 97.5 (5.0)%, and 97.6 (4.8)%. The mean ITRs achieved with the binary pattern were 64.8 (8.8), 63.7 (11.5), and 59.5 (12.5) bits/min; the mean ITRs achieved with the quintary pattern were 63.9 (6.1), 59.2 (11.4), and 55.9 (14.8) bits/min. On average, the spelling times for the binary pattern were 45.2 (7.0), 46.4 (9.8), and 50.1 (12.5) s; the spelling times for the quintary pattern were 45.3 (4.4), 50.7 (12.0), and 59.7 (37.8) s.  Figure 7 shows the achieved ITRs per flickering pattern. Regarding the differences between the binary and quintary patterns per refresh rate, analysis with paired *t*-tests did not reveal statistically significant differences (*p* > 0.05) for neither the accuracy nor the ITR.

### 3.3. Questionnaire Results

**Figure 8:**
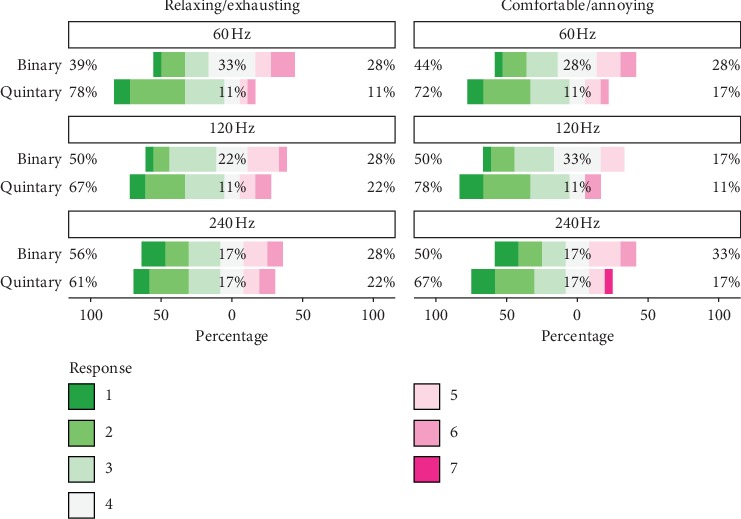
Questionnaire results. Responses from all 18 participants regarding visual stimulation were given on the basis of a 1–7 Likert scale.

Figure 8 summarizes the questionnaire responses. Regarding the first question (relaxing/exhausting), the median ratings for the binary pattern were 4, 3.5, and 3 for the 60 Hz, 120 Hz, and 240 Hz update rates, respectively; the median ratings for the quintary pattern were 2.5, 3, and 3.

The medians of the binary and quintary patterns were significantly different only for the 60 Hz setup; the *p* values of Wilcoxon signed-rank tests were 0.003, 0.065, and 0.67 for 60, 120, and 240 Hz, respectively. According to the Friedman analysis, the differences between refresh rate settings were not significant for the binary (*p* > 0.05) and quintary (*p* > 0.05) patterns.

Regarding the second question (comfortable/annoying), the median ratings for the binary pattern were 4, 3.5, and 3.5, and for the quintary pattern, the ratings were 3, 2.5, and 3.

Again, only for the 60 Hz comparison, the medians of binary and quintary patterns were significantly different; the *p* values of Wilcoxon signed-rank tests were 0.009, 0.084, and 0.077 for 60, 120, and 240 Hz, respectively. According to the Friedman analysis, the differences between refresh rate settings were not significant for the binary (*p* > 0.05) and quintary (*p* > 0.05) patterns.

We further grouped the scores into relaxing (1–3), neither relaxing nor exhausting (4), and exhausting (5–7). Analogously for the second question, we grouped the scores into comfortable (1–3), neither comfortable nor annoying (4), and annoying (5–7). For all refresh rate setups, the quintary pattern was rated less exhausting and less annoying. The quintary pattern at 60 Hz was rated the least exhausting; only two out of the eighteen participants (i.e., 11%) found this flickering design exhausting. The binary pattern was rated exhausting by four participants (28%) for all refresh rates. Regarding the second question, the quintary pattern at 120 Hz was rated least annoying (two out of eighteen, i.e., 11%).

Overall, answers indicate that the quintary flickering patterns are perceived as less annoying. According to additional comments from the participants, with the quintary pattern, it was easier to focus on the target letters. One participant found that the 60 Hz binary pattern caused headaches during the training stage. Several participants commented that the quintary flickering was less fatiguing.

## 4. Discussion

The aim of the study was to explore more user-friendly flickering patterns for c-VEP-based BCIs. Two flickering patterns, binary and quintary *m*-sequences, were tested with different flickering speeds. Both code sequences are orthogonal to their time lags. While the binary *m*-sequence is well established in BCI research, the quintary *m*-sequences have so far not been tested. Due to the nonlinearity of the visual system (e.g., due to bifurcation or period-doubling), the elicited responses obtained by visual stimulation with the orthogonal patterns have nonorthogonal autocorrelations (see, e.g., [23]). Previous studies with online BCI systems showed that the accuracies obtained with *m*-sequence-based flickering patterns are nonetheless quite high [5, 28, 37]. In a previous study, we compared SSVEP and c-VEP flickering patterns. It was observed that the latter yielded on average higher offline accuracies [25].

The acceptance of BCIs based on visual evoked potentials may depend on two factors, the user friendliness and the BCI performance. A major focus of this study was on the aspect of user friendliness. The presented quintary sequence allowed for a more subtle stimulation in comparison with the conventionally used binary pattern and was rated as slightly more user-friendly according to our questionnaire.

The stimulus colour is a key parameter for BCIs based on visual stimulation. In this study, black and white or different grey shades were used for the binary and quintary stimulus patterns, respectively. Humans have different responses to stimuli of different colours. The human retina contains two types of photoreceptors, rods and cones. The rod cells are responsible for black-and-white vision at low light levels; the cones are responsible for colour vision. There are three subtypes of cones that reflect the response to various wavelengths of light, blue cones, green cones, and red cones. As noted by Wei et al. [6], white colour stimulates all three types of cones, and therefore, it may lead to the strongest VEP response. Aminaka et al. [38] implemented a c-VEP flickering paradigm with four green and blue chromatic flashing targets in order to reduce the risk of photosensitive epilepsy. In terms of performance, the authors did not observe any significant differences in the conventional black and white flashing pattern. Instead of different shades of grey, the digits of the *m*-sequence could be encoded with different colours, stimulating the different types of cones.

In addition to the colour of the targets, the flickering speed impacts the load on the visual channel. While high-frequency systems are less fatiguing, they tend to yield lower selection speeds. In this study, eight targets were used, which is a comparably low number for c-VEP studies. Still, due to the low classification time windows employed, ITRs between 55 and 65 bits/min were achieved with the different flickering modalities.

The achieved ITRs are slightly higher than those in low-target high-frequency SSVEP BCIs: Armengol-Urpi and Sarma [18] reported a mean ITR of 15.7 bits/min for strong flickering and 13.6 bits/min for weak flickering using a four-target SSVEP system with frequencies ranging from 40 to 45 Hz in a virtual reality application. Jiang et al. [19] reported a mean ITR of 18.8 bits/min in an online experiment using a 4-target system with phase-shifted 60 Hz stimuli.

Recently, a multitarget c-VEP system with fast flickering speed was tested: Başaklar [3] implemented a 36-target c-VEP system employing a 127 bit *m*-sequence at refresh rates of 60 Hz, 120 Hz, and 240 Hz. The authors reported average ITRs and accuracies of 85.9 bits/min and 92% for 60 Hz, 94.2 bits/min and 97% for 120 Hz, and 78.7 bits/min and 87% for 240 Hz. The authors concluded that the 120 Hz refresh rate setup is best to use in multitarget BCIs, whereas the 240 Hz refresh rate may be a good choice for low-target systems. Indeed, in this study, the differences in BCI performance between the tested patterns were not significant. According to the within-subject comparison, the tested flickering patterns were equally effective. Further tests of the quintary pattern with multitarget systems are planned.

## 5. Conclusions

This study explored the usage of quintary *m*-sequences for BCIs based on c-VEPs. The conventional binary and the proposed quintary patterns were compared in an online spelling experiment with different refresh rate setups. In terms of user friendliness, we found that the quintary pattern was perceived as more comfortable and relaxing than the binary pattern. Especially, the typically used binary 60 Hz pattern was perceived as annoying by more than a quarter of the participants. In terms of BCI performance, no significant differences between the patterns were found, suggesting that further c-VEP experiments could be designed with the proposed quintary pattern.

## Figures and Tables

**Table 1:** Summary of the mean (SD) or median values from the offline analysis, online performance, and questionnaire per flickering pattern across 18 participants.

	60 Hz		120 Hz		240 Hz	
	Binary	Quintary	Binary	Quintary	Binary	Quintary
Offline accuracy (%)	97.7 (2.8)	98.7 (3.1)	99.0 (2.3)	96.9 (9.8)	94.7 (9.6)	96.3 (12.4)
Online accuracy (%)	99.4 (1.8)	98.5 (2.5)	97.6 (6.0)	97.5 (5.0)	97.9 (3.6)	97.6 (4.8)
Experiment time (s)	45.2 (7.0)	45.3 (4.4)	46.4 (9.8)	50.7 (12.0)	50.1 (12.5)	59.7 (37.8)
ITR (bits/min)	64.8 (8.8)	63.9 (6.1)	63.7 (11.5)	59.2 (11.4)	59.5 (12.5)	55.9 (14.8)
Relaxing/exhausting	4	2.5	3.5	3	3	3
Comfortable/annoying	4	3	3.5	2.5	3.5	3

The provided values for the offline accuracies were achieved with a classification time window of 1 s.

## Data Availability

The recorded data sets cannot be shared according to legal guidelines. All participants were informed that the information needed for the analysis of the experiments was stored anonymously and will be deleted after a certain time period.
